# Rhabdomyolysis Associated With Febuxostat in a Non-Chronic Kidney Disease (CKD) Patient: Diagnostic and Management Challenges

**DOI:** 10.7759/cureus.63303

**Published:** 2024-06-27

**Authors:** Armend Balidemaj, Samer Kholoki

**Affiliations:** 1 Internal Medicine, Saint James School of Medicine, The Valley, AIA; 2 Internal Medicine, La Grange Memorial Hospital, Chicago, USA

**Keywords:** myopathy, gout management, xanthine oxidase inhibitor, drug-induced myopathy, febuxostat, rhabdomyolysis

## Abstract

Rhabdomyolysis is a critical medical condition characterized by the rapid breakdown of muscle tissue, releasing substances such as myoglobin and creatine kinase into the bloodstream, potentially leading to acute kidney injury. The etiology includes trauma, exertion, genetic factors, infections, and adverse drug reactions. Febuxostat, a non-purine selective xanthine oxidase inhibitor used to manage hyperuricemia in gout patients, is typically well-tolerated but has been associated with rare instances of severe adverse reactions like rhabdomyolysis. This case report describes a case of a 73-year-old male who developed rhabdomyolysis shortly after initiating febuxostat for gout management. He presented with significant lower back pain and progressive leg weakness, initially suspected to be an exacerbation of his degenerative disk disease. Laboratory findings revealed alarmingly elevated creatine phosphokinase levels, and diagnostics excluded other potential etiologies. The patient's condition significantly improved following the cessation of febuxostat and initiation of supportive care, including high-dose intravenous corticosteroids and hydration. This case underscores the need for vigilance in monitoring for rhabdomyolysis in patients starting febuxostat, especially when presenting with unexplained muscle weakness or pain.

## Introduction

Rhabdomyolysis is a severe condition characterized by the rapid breakdown of skeletal muscle tissue, releasing myoglobin, creatine kinase, and electrolytes into the bloodstream, potentially leading to acute kidney injury [1]. The etiology of rhabdomyolysis is broad, including traumatic, exertional, genetic, infectious, and toxic causes, such as adverse drug reactions [2]. It can serve as an early indicator of underlying genetic or metabolic conditions, emphasizing the importance of recognizing its potential as a sign of more serious diseases [3]. Understanding the pathophysiology and symptom severity is crucial for timely diagnosis and treatment initiation to prevent complications such as multi-organ failure and death [4]. Prompt identification and appropriate management are vital to reduce the risk of acute kidney injury and other life-threatening outcomes associated with rhabdomyolysis [5].

In recent years, the association between certain medications and the onset of rhabdomyolysis has garnered significant clinical attention. Febuxostat, a non-purine selective inhibitor of xanthine oxidase, is commonly prescribed for the management of hyperuricemia in patients with gout. It offers an alternative to allopurinol, especially for patients who have intolerance or inadequate response to the latter. Since its approval, febuxostat has been generally well-tolerated, with common side effects including liver enzyme elevations, nausea, and arthralgia [6]. Isolated cases of severe adverse reactions of febuxostat, such as rhabdomyolysis, have been reported [7], although they remain rare. Studies have explored the association between febuxostat and muscle injury, with findings suggesting a potential risk increase [8].

The prevalence of drug-induced rhabdomyolysis varies among different studies. One study found a prevalence of 7.2% in poisoned hospitalized patients, with the most common cause being methadone poisoning [9]. Another study reported a prevalence of 7% among patients hospitalized for acute poisoning, with narcotic drugs being the main reason for rhabdomyolysis development [10]. Additionally, a study focusing on statin-induced rhabdomyolysis highlighted the importance of drug interactions, especially with CYP3A4 inhibitors like ciprofloxacin and amiodarone [11]. Furthermore, a study exploring the association between statin use and rhabdomyolysis found that high-dose lipophilic statins in combination with certain drugs significantly increased the odds of rhabdomyolysis [12]. Overall, these studies emphasize the significance of monitoring and managing drug-induced rhabdomyolysis, especially in cases involving specific drug interactions.

The case presented herein involves a 73-year-old male who developed severe rhabdomyolysis shortly after initiating treatment with febuxostat. This case is particularly instructive for the clinical community due to the rarity of this adverse effect in association with febuxostat. It underscores the critical need for healthcare providers to be vigilant when prescribing new medications, particularly in older adults who may have multiple comorbidities. Monitoring for unusual symptoms, especially when a new medication is started, is paramount to prevent severe complications.

## Case presentation

A 73-year-old gentleman who had been enjoying a vacation involving fly fishing presented to the clinic with significant lower back pain and progressive leg weakness, which he initially thought were due to an aggravation of his existing degenerative disc disease. This progressive weakness was characterized by difficulty in ascending and descending stairs and arising from chairs, symptoms that had developed over the week prior to his presentation.

Crucially, the patient had started on a new medication, Uloric (febuxostat) 80 mg daily, approximately one month before these symptoms appeared. This new treatment was prescribed for gout management, replacing his previous medication regimen.

Upon examination, the patient exhibited pronounced proximal myopathy, with Grade 2 power in hip flexion. Initial laboratory tests indicated alarmingly elevated creatine phosphokinase (CPK) levels at approximately 50,000 U/L and abnormal liver function tests, prompting immediate hospital admission for suspected myositis. Further diagnostic workup during his hospital stay was performed (Table 1).

**Table 1:** Laboratory testing results on admission and on day 7 of treatment

Labs Variable	Day 1	Follow-up (7 days after admission)	Reference Range
Blood Urea Nitrogen (BUN), mg/dL	89	28	8 - 20
Creatinine (Cr), mg/dL	2.71	0.74	0.8 - 1.3
Sodium (Na), M Eq/L	141	138	136 - 145
Potassium (K), M Eq/L	4.2	4.1	3.8 - 5.2
Chloride (Cl), M Eq/L	102	100	98 - 107
Calcium (Cl), mg/dL	8.1	7.9	8.5 - 10.2
Phosphorus (P), mg/dL	4.6	4.2	2.8 - 4.5
Magnesium (Mg), mg/dL	1.6	1.6	1.7 - 2.2
Creatinine Phosphokinase (CPK), U/L	55,182	2,762	M: 55 - 170, F: 30 - 140
Erythrocyte Sedimentation Rate (ESR), adult, mm/h	43	26	M: 0 - 5, F: 0 - 20
Aspartate Aminotransferase (AST), adult, U/L	2,748	289	10 - 40
Alanine Aminotransferase (ALT), adult, U/L	1,299	878	10 - 40

Imaging studies reinforced the absence of acute structural causes for the patient’s symptoms. Renal ultrasound showed no abnormalities such as cortical thinning, hydronephrosis, or renal calculi, with kidneys measuring normal sizes and normal bladder activity. The liver ultrasound revealed a homogeneous echotexture with no hepatic masses or dilated ducts, and normal flow within the hepatic and portal veins, effectively ruling out structural liver disease.

Electromyography (EMG) testing showed severe loss of voluntary motor units in the right quadriceps muscle group and less pronounced involvement on the left side, consistent with myopathy rather than nerve damage. A muscle biopsy of the left quadriceps confirmed necrotizing myopathy.

Given the timing of symptom onset following the initiation of Uloric and the exclusion of other potential neurological or metabolic etiologies through extensive diagnostics, a strong suspicion of drug-induced rhabdomyolysis was considered. The patient was treated with high-dose intravenous methylprednisolone 40 mg for three days and aggressive hydration. Physical therapy was also initiated to regain muscle strength.

Significant improvement was noted seven days after the discontinuation of Uloric; CPK levels reduced to 2,762 U/L, liver enzymes continued to trend downwards (Table 1), and renal function restored to baseline levels prior to discharge 19 days later. These clinical and laboratory improvements following the withdrawal of Uloric and the introduction of corticosteroids provided compelling evidence linking febuxostat to the onset of rhabdomyolysis in this patient.

## Discussion

Rhabdomyolysis is a serious condition, marked by the dissolution of skeletal muscle integrity secondary to injury [10] that can be induced by a variety of drugs and substances, including opioids, alcohol, poisons, and several classes of prescription medications. Studies, such as those by Nahandi et al., have identified common culprits including antiepileptics, antipsychotics, antihistamines, and notably statins, which are frequently reported triggers due to their widespread use [9,13,14]. Substances such as alcohol, narcotics, and psychotropic drugs have also been implicated in cases of rhabdomyolysis, as they can directly cause muscle toxicity, disrupt muscle metabolism, or create electrolyte imbalances [9]. Notably, beta-blockers such as propranolol, labetalol, pindolol, and xamoterol, and rarely nebivolol, have also been associated with rhabdomyolysis [15,16]. These findings underscore the diverse range of medications that can potentially lead to this serious condition.

Drug-induced rhabdomyolysis can result from direct muscle toxicity, disturbed muscle metabolism, or electrolyte imbalances, as highlighted in various studies [17]. Rhabdomyolysis severity can indeed differ based on the causative substance. Psychotropic substances tend to result in more severe cases compared to chemical substances. Research shows that patients intoxicated with psychotropic substances exhibit significantly more common rhabdomyolysis symptoms than those intoxicated with chemical substances [10]. Factors like high dosage, renal and hepatic impairment, and genetic variants can increase the risk of statin-induced myopathy, emphasizing the importance of multifactorial risk assessment in high-risk patients [18]. Therefore, a proactive approach to assessing risk factors and potential drug interactions is crucial in guiding individualized statin therapy and preventing severe adverse reactions like rhabdomyolysis.

Research has shown that patients with significantly decreased estimated glomerular filtration rate (eGFR) face a heightened risk of developing myopathy when treated with febuxostat. It is recommended that creatine kinase levels be routinely monitored to facilitate the early identification of myopathy associated with febuxostat use, especially in individuals with chronic kidney disease [19]. However, findings from a systematic review and meta-analysis present contrasting results, suggesting that febuxostat does not significantly impact the risk of muscle injury. Mitsuboshi and Kotake discovered that two out of three documented case reports of rhabdomyolysis induced by febuxostat involved patients with chronic kidney disease. Additionally, an association between the use of febuxostat and the development of myopathy has been noted in patients with chronic kidney disease [8]. These findings underscore the need for further high-quality observational studies that include long-term follow-up to clarify the relationship between febuxostat and muscle injury risks

Two noteworthy cases illustrate the potential for febuxostat-induced rhabdomyolysis in a patient with chronic kidney disease. These cases were successfully managed by discontinuing the medication and applying conservative therapy [7,20]. But our case contrasts with previous cases where chronic kidney disease was present, further making it difficult to understand the mechanism of febuxostat causing rhabdomyolysis.

## Conclusions

This case report highlights the critical need for awareness among healthcare providers about the risk of rhabdomyolysis associated with febuxostat, even in patients without chronic kidney disease. The temporal correlation between the onset of symptoms and the initiation of febuxostat treatment, alongside the significant improvement post-discontinuation, underscores the potential for this drug to cause severe muscle injury. This report serves as a crucial reminder for clinicians to monitor for signs of muscle weakness and to evaluate creatine kinase levels when patients begin treatment with febuxostat. Early intervention, including the cessation of the drug and supportive care, is essential to mitigate adverse outcomes and ensure patient safety.
